# Detecting Occult Sentinel Node Metastases in HNSCC: The Emerging Role of lncRNAs as Biomarkers and Future Perspectives for USgFNAB Molecular Profiling

**DOI:** 10.3390/cancers18030427

**Published:** 2026-01-28

**Authors:** Boštjan Lanišnik, Janez Mohorko, Uroš Potočnik

**Affiliations:** 1 Department of Otorhinolaryngology, Head and Neck Surgery, Maribor University Medical Centre, 2000 Maribor, Slovenia; 2 Centre for Human Molecular Genetics and Pharmacogenomics, Faculty of Medicine, University of Maribor, 2000 Maribor, Slovenia; uros.potocnik@um.si

**Keywords:** head and neck squamous cell carcinoma, cervical lymph node metastasis, long non-coding RNA, ultrasound-guided fine-needle aspiration biopsy, molecular diagnostics, biomarker discovery

## Abstract

**Background**: Accurate detection of cervical lymph node metastases is a critical determinant of prognosis and treatment planning in head and neck squamous cell carcinoma (HNSCC). Although ultrasound-guided fine-needle aspiration biopsy (USgFNAB) is widely used as a minimally invasive diagnostic tool, its sensitivity for detecting occult metastases remains limited. Current preoperative staging modalities are further constrained by operator dependency and suboptimal specificity in early-stage disease. Integration of molecular diagnostics, particularly the analysis of long non-coding RNAs (lncRNAs), represents a promising strategy to enhance diagnostic accuracy. **Objective**: This review synthesizes the current evidence on lncRNA expression profiles in HNSCC, with an emphasis on their association with lymph node metastasis and potential application in FNAB-derived material for pre-treatment staging. **Methods**: A structured literature search was conducted, focusing on studies evaluating lncRNA expression profiles in HNSCC and their relevance to lymph node metastasis, with a particular focus on the feasibility of analysis of USgFNAB samples. **Results**: Multiple lncRNAs, including HOTAIR, MALAT1, UCA1, TUG1, AFAP1-AS1, H19, MEG3, and ADAMTS9-AS2, have been implicated in metastatic progression through their involvement in diverse mechanisms such as epithelial-to-mesenchymal transition, chromatin remodeling, angiogenesis, and pre-metastatic niche formation. Elevated expression of several of these transcripts correlates with adverse clinicopathological features, including advanced tumor stage, extranodal extension, and reduced survival. However, no studies have profiled lncRNA expression in matched primary tumors and metastatic lymph nodes, and transcriptomic analysis of FNAB samples remains largely unexplored in HNSCC. **Conclusions**: lncRNAs represent promising molecular biomarkers for enhancing the sensitivity and specificity of USgFNAB in detecting occult cervical metastases. Future research should prioritize paired tumor–node lncRNA profiling, validation of FNAB-based molecular assays, and integration of multi-omics data for predictive modeling. Overall, integrating lncRNA analysis into ultrasound-guided fine-needle aspiration biopsy may enhance the detection of occult nodal metastases in head and neck squamous cell carcinoma and support more accurate nodal staging in clinically node-negative patients.

## Simple Summary

The spread of head and neck cancer to lymph nodes in the neck strongly affects treatment decisions and patient outcomes, yet current diagnostic methods often fail to detect small or early metastases before treatment begins. Ultrasound-guided fine-needle aspiration biopsy is a widely used, minimally invasive test, but its ability to detect hidden metastases is limited. Recent research shows that long non-coding RNAs, a class of stable genetic molecules involved in cancer progression, may serve as sensitive molecular markers of metastatic disease. This review summarizes the current evidence on the role of these molecules in head and neck cancer and discusses how their analysis in biopsy samples could improve the detection of occult lymph node metastases. Integrating molecular biomarkers with existing diagnostic protocols has the potential to reduce unnecessary surgery, improve treatment planning, and ultimately enhance patient outcomes.

## 1. Introduction

USgFNAB is a clinically well-established, minimally invasive technique for obtaining cellular material from cervical lymph nodes, which is particularly useful for pre-treatment staging of the neck in early HNSCC. The presence of neck metastasis is crucial when determining the treatment plan, as regional nodal involvement represents a significant prognostic factor for survival. Current preoperative imaging methods often miss early-stage occult metastases due to limited sensitivity and specificity. Histopathological examination following surgical removal of the specimen has significant drawbacks and is only available for postoperative staging. This information is unavailable if the patient is treated with a non-surgical modality. USgFNAB, aided by cytopathological immunostaining methods, provides a minimally invasive alternative with almost 100% specificity, although its sensitivity remains approximately 50%. The efficacy of the technique is limited by the proficiency of the physician performing the FNAB, the experience of the cytopathologist, and the possibility of sampling error, but it offers advantages such as low cost, no radiation exposure, and wide availability. The use of quantitative molecular assessments, such as qRT-PCR assays and the analysis of lncRNAs, offers promising avenues for enhancing diagnostic accuracy. LncRNAs, which play essential roles in gene regulation and tumorigenesis, are emerging as promising molecular biomarkers in cancer. This review explores the existing literature on lncRNA expression profiles in HNSCC, with a focus on comparisons between primary tumors and associated lymph node metastases. Studies have identified specific lncRNAs, such as HOTAIR, UCA1, MALAT-1, AFAP1-AS1, and ADAMTS9-AS2, that are associated with advanced stages of laryngeal, oral cavity, and nasopharyngeal carcinoma (NPC). These lncRNAs are involved in regulating various biological processes, such as proliferation, migration, invasiveness, and apoptosis, and have been correlated with tumor characteristics, the presence of lymph node metastases, and prognosis. The mechanism(s) of tumor spread to sentinel lymph node is imperfectly understood. Recent evidence suggests that the primary tumor may prime the draining lymph node for metastatic spread. It releases soluble factors and extracellular vesicles that are transported to the draining lymph node, where they participate in the creation of a so-called pre-metastatic niche, forming a suitable microenvironment and enabling metastatic spread to the lymph node [1]. This mechanism has not yet been validated in HNSCC, and sensitive biomarkers are needed to identify patients in whom a pre-metastatic niche is developing.

## 2. Material and Methods

This narrative review was conducted through a comprehensive literature search aimed at identifying studies relevant to the role of lncRNAs in HNSCC, with a particular focus on lymph node metastasis, USgFNAB, and molecular diagnostic applications. The literature search was performed using the Web of Science, ScienceDirect, and ProQuest Dissertations & Theses Global databases. The search covered publications from January 2000 to December 2025 and employed combinations of the following keywords: “long non-coding RNA”, “head and neck cancer,” and “ultrasound-guided fine-needle aspiration biopsy.”; the keywords were applied individually and in Boolean combinations No formal exclusion criteria were applied in order to capture a broad overview of experimental, translational, and clinical research relevant to the topic. Original research articles, reviews, and selected preclinical and translational studies were included where they contributed to understanding lncRNA biology, metastatic mechanisms, or molecular analysis from biopsy-derived material. While searches using the individual terms retrieved numerous records, combining lncRNAs with head and neck cancer markedly reduced the number of relevant publications, and no studies were identified that simultaneously addressed lncRNAs, ultrasound-guided FNAB, and head and neck cancer, highlighting a clear gap in the current literature. The identified literature was subsequently synthesized thematically across all sections of the manuscript, including biological background, translational considerations, and future research directions.

## 3. Current Gold Standard of Early Head and Neck Squamous Cell Carcinoma Diagnostic and Management Protocols

It is widely accepted that regional LN metastasis is the single most important prognostic factor in HNSCC [2,3]. The American Joint Committee on Cancer (AJCC) acknowledges this view, as demonstrated by their recommendation for tumor upstaging in cases with node-positive (N+) neck [4]. Therefore, neck nodal staging is paramount in treatment planning. The main controversy is the approach to treating early-stage head and neck cancer patients with clinically N0 neck. Regardless of the modality, there are two options: elective treatment of the regional metastases (radiotherapy or neck dissection) or watchful waiting. Clinical N0 staging is determined through different imaging modalities as well as USgFNAB. Because of the low sensitivity of all these methods, neck nodes are electively treated if there is a 20% or more chance of occult metastases; this cut-off was determined based on historical retrospective studies [5,6]. In patients with N+ neck, neck nodes and metastases are either removed surgically (neck dissection) in primary surgical therapy or treated with primary chemo/radiation. Radiotherapy with or without chemotherapy is also used as a postoperative adjuvant treatment if adverse features (e.g., extranodal extension of the metastasis) are present. There is a general consensus that a salvage neck dissection is indicated for patients with a primary tumor that showed a good response to a non-surgical modality but with residual and still operable neck metastasis [7]. Neck dissection has considerable quality-of-life implications for patients because it is a debilitating procedure with potential complications such as shoulder dysfunction, myofascial pain, or even death. Currently, there are no reliable imaging techniques for identifying occult metastases. PET/CT exhibits the highest sensitivity and negative predictive value but has the lowest specificity and accuracy [8]. Due to its high negative predictive value, it is useful in patient follow-up after primary chemoradiotherapy to prevent unnecessary neck dissection [9]. On its own, PET/CT is not sufficient for preoperative diagnosis because it produces many false positives. US, CT, and MRI offer good spatial resolution and are needed to improve diagnostic accuracy.

## 4. Sentinel Node Biopsy

In an effort to minimize the impact of the surgical therapy for N0 neck, some doctors use the sentinel node technique to identify the first draining node; however, in HNSCC, the use of this technique has not been uniformly accepted. This technique was also studied at our institution and we did not find a reliable method to intraoperatively identify metastases; however, no attempt was made to preoperatively identify occult metastases [10]. It has been shown that HNSCC has a predictable pattern of spreading in the neck, with the initial involvement of the first nodal echelon and later progressing more distally to lower neck regions. This fact is the basis for implementing sentinel node biopsy [11,12,13]. In unilateral neck disease, contralateral metastases are rare and usually involve neck levels II and III. Recent studies found that the incidence of contralateral metastases is around 6% [14,15]. The principle of sentinel node biopsy is based on predictable lymph drainage from the HNSCC. The sentinel node is the most proximal draining node and is the first node to be targeted by metastatic cells. Therefore, the status of the sentinel node should reflect the stage of disease in the neck [16]. The procedure starts with preoperative injection of the radiotracer Technetium 99m (99mTc) around the tumor bed, followed by sentinel node localization with lymphoscintigraphy. Intraoperatively, the sentinel node is identified and removed with the help of a gamma probe. The node is sent to the pathologist for serial sectioning. If metastasis is confirmed, a formal delayed neck dissection is warranted. If the sentinel lymph node is negative, no further dissection is needed and the patient is spared from further surgery [17]. According to the latest meta-analyses, SLNB (sentinel lymph node biopsy) shows good sensitivity and specificity [18,19,20,21,22]. Following these findings, the SLNB protocol has been incorporated into the NCCN guidelines for early-stage oral cavity squamous cell carcinoma (SCC) [7]. SLNB has an overall sensitivity of 86% and NPV of 95% [17]. Despite this, SLNB has not gained widespread use, mainly due to logistical problems related to the preoperative radiotracer injection, patient and surgeon exposure to radiation, need for lymphoscintigraphy, and time requirements for intraoperative serial sectioning of the removed lymph node. Numerous studies evaluated various molecular techniques for intraoperative lymph node assessment. For instance, the molecular markers pemphigus vulgaris antigen and epithelial cell adhesion molecule were used in a multiplexed qRT-PCR assay for intraoperative evaluation of lymph nodes, and the results were compared to those obtained using frozen sections and immunohistochemistry. This technique showed 92% sensitivity and 96% specificity for detecting lymph node metastasis. The authors expect that this technique can also be used with FNAB [23,24]. Intraoperative FNAB with flow cytometry was also evaluated as a proof of concept for rapid intraoperative nodal staging. Using anti-human cytokeratin 5/8, EpCAM, and mucin 1, 3, which are well-established antibodies for the detection of HNSCC, an NPV of 99.97% was achieved [25]. However, intraoperative lymph node assessments require surgery, which puts the patient at risk for complications and cannot be used for pre-treatment staging of HNSCC.

## 5. Ultrasound-Guided Fine-Needle Aspiration Cytology

Another method for preoperative evaluation of small neck lymph nodes with a normal appearance is the addition of fine-needle aspiration cytology to neck US (USgFNAB). Neck US is a standard imaging method in the diagnostic protocol for head and neck malignancies. In the case of clinically N0 neck, a FNAB of the first echelon lymph node (sentinel lymph node) under US guidance can be performed. The cytologic aspirate is examined by a cytopathologist under a light microscope. To increase accuracy, cytopathologic immunostaining methods are employed. The current specificity of USgFNAB for occult neck metastasis is between 92 and 94.4%; false-positive results are rare but the sensitivity is only around 50% [26]. Limitations of USgFNAB stem from the fact that its accuracy is highly operator- and cytopathologist-dependent and there is always the possibility of a sampling error [24]. On the other hand, the advantages of USgFNAB (i.e., its low cost, no radiation exposure, and widespread availability) outweigh these limitations. The use of quantitative molecular assessment of lymph node aspirates may help to improve the diagnostic yield. However, translating lncRNA assays to USgFNAB material introduces several practical challenges that can affect analytical sensitivity and reproducibility. FNAB specimens often contain low and variable RNA yields due to limited cellularity, sampling heterogeneity, and rapid RNA degradation in RNase-rich environments. The aspirate frequently includes abundant non-tumor components (blood, lymphoid cells, and stromal elements, etc.) which can dilute the tumor fraction and obscure metastasis-specific lncRNA signals, particularly in micrometastatic disease. Pre-analytical factors such as needle gauge, number of passes, aspiration technique, specimen handling time, and the choice of preservative (e.g., direct smear fixation vs. liquid-based cytology media) further contribute to sample variability and batch effects. These limitations underscore the need for standardized processing workflows, cell-content assessment, and robust normalization strategies when developing FNAB-based lncRNA biomarker panels for clinical staging. This is already in use in metastatic human papilloma virus (HPV)-positive HNSCC. FNAB supernatant has been shown to be a rich source of tumor DNA that can be used to detect HPV status [27]. However, this is of limited value because only a portion of HNSCC is HPV-induced. There are only scarce reports of the use of molecular biomarkers in preoperative diagnostics of sentinel lymph nodes. If a reliable molecular biomarker for metastatic cancer cells in FNAB samples from a sentinel lymph node could be identified, it could improve pretreatment sensitivity as well as specificity in the detection of occult metastases.

## 6. Finding Biomarkers

### 6.1. Transcriptome Studies in HNSCC

#### 6.1.1. Primary Tumor Biopsies and Healthy Tissues

The genetic basis behind the development and progression of HNSCC includes extensive transcriptome changes and their associated aberrant RNA levels. Additionally, important risk factors for the development of HNSCC include HPV infection and tobacco and alcohol use. Many research groups have performed transcriptome analyses on HNSCC tissues and identified biological, prognostic, and predictive molecular signatures [28]. However, a fundamental understanding of the genetics and mechanisms implicated in HNSCC metastasis remains incomplete. cDNA microarray analyses have revealed key genes and processes, such as digestion and remodeling of the extracellular matrix (ECM), lymphocyte activation, antigen presentation, and angiogenesis, that are implicated in invasion and progression in HNSCC [29]. This has been confirmed by an integrative multi-omics study that involved DNA copy number, transcriptome, and methylome analyses of metastasizing and non-metastasizing HNSCC tumors [30]. Furthermore, HNSCC metastatic potential was assessed by comparing gene expression in tumor tissues from patients with or without lymphatic metastasis, which revealed Raf-1 as an independent prognostic risk factor for lymphatic metastasis in hypopharyngeal carcinoma [31]. To investigate the immune response to the presence of cancer, Yao et al. analyzed the immune-related prognostic biomarkers in HNSCC patients using high-throughput RNA sequencing of HNSCC tumors and matched adjacent tissues [32]. They identified 248 immune-related genes that were differentially expressed in paired tissues, 4 of which (PVR, TNFRSF12A, IL21R, and SOCS1) were significantly associated with overall survival. A few studies analyzed regulatory RNA signatures in HNSCC. For instance, by examining the next-generation RNA-seq data from tumors and matched normal tissues, Zou et al. found 2808 lncRNAs that were significantly differentially expressed; two of these lncRNAs (lnc-LCE5A-1 and lnc-KCTD6-3) were dramatically down-regulated in HNSCC and their levels negatively correlated with poor survival [33]. Although the sample size in individual transcriptomic studies is often limited, many investigations have included datasets from The Cancer Genome Atlas (TCGA) and other databases. For instance, analysis of RNA-seq and clinical data from 500 HNSCC patients from TCGA enabled the identification of a panel of six mRNAs (FRMD5, PCMT1, PDGFA, TMC8, YIPF4, and ZNF324B) that could predict patient prognosis [28]. Doh Young et al. correlated RNA-seq data of primary tongue cancer with matched clinical data obtained from TCGA for 515 patients [34]. The clinical data (including the tumor staging and metastasis data) was analyzed to investigate the transcription profiles of tongue cancer associated with early LN metastasis, which was found to be associated with overexpression of ACTA1. Furthermore, Jin and Qin carried out an integrated bioinformatics analysis of miRNAs and mRNAs in HNSCC by analyzing four independent HNSCC microarrays (comparing tumor vs. normal tissues) from the Gene Expression Omnibus, and identified upregulation of SLC16A1, COL4A1, and several other genes as important HNSCC biomarkers [35]. Irimie et al. used TCGA data of 519 HNSCC patients to identify differentially expressed genes that can stratify the patients based on their smoking status and HPV infection [36]. Using the TCGA data of 528 HNSCC tumors, Saidak et al. characterized specific gene expression changes associated with perineural invasion (PNI), which could be used for therapeutic patient stratification and prediction of recurrence risk. They reported that tumors with histological PNI had increased activation levels of the Akt/PKB and mTOR kinases [37].

#### 6.1.2. Primary Tumor Biopsies Compared to Lymph Node Biopsies

Earlier microarray studies showed that gene expression patterns in lymph node metastases (LNMs) were largely comparable to those of the corresponding primary tumors from which they arose [38]. In contrast, more recent comparisons of gene profiles from primary HNSSC and their metastases have reported specific differences in their gene expression. For instance, Claudin-7 (implicated in tight junctions in epithelial cells) is under-expressed in most LNMs compared to only 29% of the corresponding primary tumors showing under-expression. Maspin (implicated in the regulation of the actin cytoskeleton) is also under-expressed in most LNMs and over-expressed in almost 50% of the corresponding primary tumors [39]. A high-throughput investigation using next-generation sequencing of mRNAs and miRNAs from matched primary tumors and LNMs from 34 HNSCCs patients revealed that LNMs can be stratified into three subtypes: (i) an immune subtype, (ii) an invasive subtype, and (iii) a metabolic/proliferative subtype [40]. The invasive subtype was associated with significantly worse locoregional control and survival. The matched primary tumor specimens could not be stratified into these subtypes. The authors concluded that transcriptional profiles of LNMs can predict outcomes better than the transcriptional profiles of primary tumors. Puram et al. performed single-cell transcriptomic analysis (scRNA-seq) of primary-tumor and metastatic-LN biopsies from 18 HNSCC patients [41]. Malignant cells were found to vary within and between tumors with respect to the transcription of genes involved in the cell cycle, stress, hypoxia, epithelial differentiation, and partial epithelial-to-mesenchymal transition (p-EMT). Cells expressing p-EMT markers were found to localize to the leading edge of primary tumors. Importantly, p-EMT could serve as an independent predictor of nodal metastasis, grade, and adverse pathologic features. Another scRNA-seq analysis by Parikh et al., which examined expression changes in malignant cells in LNs versus primary tumors, revealed that CXCL14 was the most significantly downregulated gene among malignant cells in LNs relative to primary tumor, supporting its involvement in metastasis in oral cancer [42].

#### 6.1.3. HNSCC Lymph Node Aspirates

To date, no RNA-seq transcriptomic analyses have been performed on FNAB samples from HNSCC patients. Only a few targeted studies in other cancers analyzed the expression of individual genes in FNAB samples, including a study that detected increased expression of the TIMP1 and CHI3L1 genes in thyroid cancer patients compared to patients with benign nodules [43]. In thyroid cancer, advances in technology and the use of next-generation sequencing (NGS) of FNAB samples allowed for the identification of a set of specific miRNAs that can distinguish well-differentiated thyroid cancer from the benign thyroid nodules with 100% specificity [44]. While several oncogenic and suppressor miRNAs are associated with HNSCC, they have not yet been validated using FNAB samples [45].

#### 6.1.4. Brush Biopsies

To the best of our knowledge, neither RNA-seq transcriptome nor integrative multi-omic studies using brush biopsies of HNSCC patients have been published. A few reports have described using brush swabs to determine the RNA expression of target genes; determine the presence of microsatellite instability and HPV; examine the epithelial cytology and chromosomal aberrations; and monitor patients after treatment [46,47,48]. These studies demonstrated that brush sampling methods can effectively detect malignant and pre-malignant lesions in the oral and oropharyngeal mucosa. Liquid-based brush cytology showed high diagnostic accuracy for squamous cell carcinoma before treatment (sensitivity: up to 88%; specificity: 100%) and was particularly reliable for HPV-DNA detection. Combining oral liquid-based brush cytology with immunocytochemistry for DNA mismatch repair proteins further enhanced diagnostic precision, achieving sensitivities and specificities above 90% for discriminating oral epithelial dysplasia and carcinoma. From this, we can surmise that brush swab analysis coupled with molecular assays can provide a sensitive and specific alternative to traditional biopsies for early detection and follow-up of HNSCC. There have also been a few attempts at epigenomic and proteomic profiling of HNSCC from brush biopsies [49,50,51]. Those studies show that brush biopsies can be used as alternatives to classical biopsies for the acquisition of high-quality HNSCC material suitable for epigenomic and proteomic profiling. Viet et al. reported that brush samples yield DNA of comparable quality and quantity to tissue biopsies, retaining cancer vs. normal tissue methylation differences. Quantitative mass spectrometry-based profiling of brush biopsy specimens identified protein expression patterns that distinguished HNSCC from healthy mucosa, including dysregulated pathways involved in cell adhesion, cytoskeletal organization, and immune responses. Protein recovery from brush samples was sufficient for reproducible quantification, enabling biomarker panel development [49]. An overview of transcriptomic studies in HNSCC, including comparisons between primary tumors, normal tissues, lymph node metastases, FNAB aspirates, and brush biopsy samples, together with their methodological approaches and clinical relevance, is summarized in Table 1.

## 7. lncRNA Expression Profile in Metastatic HNSSC

Growing evidence highlights the role of lncRNAs in tumor progression, metastasis, and prognosis. Their stability in body fluids and specific expression profiles in advanced HNSCC suggest their potential as minimally invasive biomarkers. Non-coding RNAs (ncRNAs) play an important role at various levels of gene regulation, such as epigenetic regulation, genomic imprinting, X chromosome inactivation, transfer of nuclear and cytoplasmic RNA, transcription, and mRNA splicing [52]. ncRNAs can be divided into two subgroups: housekeeping ncRNAs and regulatory ncRNAs. Housekeeping ncRNAs include transfer RNAs (tRNAs), ribosomal RNAs (rRNAs), small nuclear RNAs (snRNAs); and small nucleolar RNAs (snoRNAs). These RNAs are expressed mostly constitutively and are important for normal cell function. Regulatory ncRNAs can be further divided into two subgroups: (i) short ncRNAs (microRNAs (miRNAs), small interfering RNAs (siRNAs), etc.) and (ii) long ncRNAs (antisense lncRNAs (aslncRNAs) and enhancer RNAs (eRNAs)). These genes are expressed at certain stages of development, during cell differentiation, or in response to external stimuli, which can affect the expression of other genes [53]. lncRNAs represent a group of RNA molecules that do not encode information for protein synthesis, but play key roles as regulators of gene expression and tumorigenesis [54,55]. They are involved in various diseases, including cancer, where they can act as tumor suppressors or oncogenes [52,56]. It is a heterogeneous group of non-coding polyadenylated RNAs longer than 200 nucleotides. lncRNAs are stable molecules that are present in human body fluids (blood, saliva, lymph, etc.) and are therefore relatively easy to detect in tissues and serum [57]. They can be located in cytosolic or nuclear fractions. Transcription of lncRNA is mediated by RNA polymerase II, similar to mRNA [58]. There is increasing evidence that lncRNAs play an important role in normal cell function, and their deregulation is strongly associated with tumor development, invasion, angiogenesis, metastasis, and chemo-resistance. Consequently, this suggests the possibility that they can be used as biomarkers for the early detection, prognosis, and treatment of cancer [59]. A review of the literature shows a lack of published studies comparing the lncRNA expression profile of primary HNSCC and its associated metastasis. A few studies have established a characteristic lncRNA expression profile for HNSCC with neck metastasis. These studies found that certain lncRNAs are frequently expressed in advanced laryngeal and oral cavity cancers (Table 2).

HOX antisense intergenic RNA (HOTAIR) is one of the most studied lncRNAs; it is associated with cancer development, metastasis, and poor prognosis [57,80,81]. It is a 2158-nucleotide long RNA molecule, primarily regulates gene expression at the transcriptional level via chromatin remodeling and epigenetic silencing, while its influence on translation is largely indirect, acting through interactions with microRNAs and modulation of mRNA stability [63]. It contributes to metastasis through multiple mechanisms, including epigenetic silencing, epithelial–mesenchymal transition, and promotion of tumor angiogenesis [82]. Specifically, it promotes EMT transition by downregulating epithelial markers such as E-cadherin and upregulating mesenchymal markers such as Snail, Vimentin, and Twist [61]. It recruits the enhancer of zeste homolog 2 (EZH2), a histone lysine methyltransferase, to the promoter region of E-cadherin. This leads to transcriptional repression of E-cadherin, thereby enhancing cellular migration and invasion [64]. A second mechanism of action is through chromatin remodeling. HOTAIR plays a role in gene silencing by interacting with the polycomb repressive complex 2 (PRC2) and lysine-specific demethylase 1 (LSD1), guiding these complexes to target genomic loci for chromatin remodeling and transcriptional repression. This remodeling activity silences tumor-suppressor genes and promotes the expression of metastasis-related genes [60]. A third mechanism of action of HOTAIR is through acting as a competing endogenous RNA (ceRNA), sponging tumor-suppressive miRNAs such as miR-326, which regulates the MTA2 (metastasis-associated gene 2) axis [62]. There is evidence that a gene polymorphism that affects HOTAIR expression is related to increased rate of lymph node involvement in oral squamous cell carcinoma (OSCC). Specifically, the single-nucleotide polymorphism (SNP) rs920778 in HOTAIR was associated with increased susceptibility to OSCC metastasis [65]. Overall, there is accumulating evidence that HOTAIR is highly expressed in OSCC tissues, particularly in samples with lymph node metastasis, and that high levels are associated with poorer overall survival and disease-free survival [83]. In laryngeal cancer, increased expression is correlated with TNM status, histopathological grade, and the presence of metastases [63]. In vitro studies have shown that its downregulation inhibits the proliferation of cancer cells and promotes their apoptosis [61,84]. HOTAIR function is pathologically altered in cancer of the larynx, nasopharynx, and oral cavity [66,69,85]. Metastasis-Associated Lung Adenocarcinoma Transcript 1 (MALAT1) is a >8000-nucleotide-long lncRNA transcript that was first described by Ji et al. [86]. Since then, its nuclear transcript was found to be highly conserved [87]. Studies have shown its importance in regulating chromatin modification and gene transcription, and its function as a ceRNA [67,72]. Increased expression of MALAT1 in cancer promotes proliferation and metastasis [68]. Its expression is significantly increased in OSCC and there is a correlation between its high expression and LN metastasis [66]. It has also been shown to be an unfavorable prognostic factor in laryngeal cancer, where it is associated with a higher histological grade of the disease and advanced disease, and in nasopharynx cancer, where it is also associated with advanced disease and locoregional metastases [56]. MALAT1 is a promising candidate as a potential biomarker in HNSCC, but more studies are needed to elucidate its function in cancer metastasis and progression. Urothelial cancer-associated 1 (UCA1) is a 1442-nucleotide-long RNA transcript that was initially associated with the proliferation, migration, and invasiveness of urothelial carcinoma [85]. Later, studies on carcinoma of the tongue showed elevated expression of UCA1, especially in patients with LN involvement. Additional in vitro investigations of the function of this transcript showed that its elevated expression positively correlates with the ability of cancer cells to migrate, but does not affect tumor size or proliferation [69]. TUG1 (taurine upregulated gene 1) has been shown to regulate cell proliferation, invasion, and metastasis [88,89]. It is highly expressed in OSCC cells, acting as an enhancer of migratory potential of tumor cells [71]. Overexpression leads to increased metastasis of cancer cells, and knockdown suppresses cancer invasion potential [70]. Actin filament-associated protein 1 antisense RNA1 (AFAP1-AS1) is a 6810 bp long lncRNA that has been associated with many cancers [72]. Recent studies found that it influences the proliferation and metastasis of laryngeal cancer and is associated with poor histopathological characteristics of the tumor. A higher level of expression was found in oral cavity tumors than in healthy oral cavity mucosa, and elevated expression was associated with LN metastases and worse prognosis [73]. Using it as a serum biomarker for the detection of NPC was also proposed [67]. H19 is recognized as an oncogenic lncRNA, promoting metastasis through its regulatory interactions with various molecular pathways [90]. In NPC, H19 overexpression is linked to poor prognosis [76]. Overexpression in OSCC was corelated with higher rate of LN involvement [75]. It was also shown that it promotes OSCC migration and invasion through miR-138 sponging [74]. Maternally expressed 3 (MEG3) is a tumor-suppressor lncRNA that is frequently downregulated in many cancers, including OSCC. It has a role in inhibiting OSCC progression by suppressing metastasis, migration, and invasion. Low expression of MEG3 is associated with increased migration and invasion, higher rates of LN metastasis, and poorer prognosis in OSCC patients [77,78]. ADAM metallopeptidase with thrombospondin type 1 motif, 9 antisense RNA 2 (ADAMTS9-AS2) is the least studied lncRNA in head and neck tumors. Increased expression of this lncRNA in tongue cancer is associated with lymphatic metastasis and poor prognosis. Furthermore, knockdown of this gene in in vitro and in vivo studies led to inhibition of tumor growth and reduced migration and invasiveness [79].

## 8. Targets for Research

The current evidence highlights the gaps in knowledge and practice and offers clear directions for future investigations aimed at improving the detection and prognostic assessment of metastatic disease in HNSCC. In particular, the integration of molecular biomarkers—specifically lncRNAs—into pre-treatment staging protocols represents a promising avenue. Despite the extensive literature on lncRNA dysregulation, there are no published studies that directly compare lncRNA expression profiles of matched HNSCC primary tumors and corresponding metastatic cervical lymph nodes. Paired transcriptomic analyses, using both bulk RNA sequencing and single-cell RNA sequencing, could reveal metastasis-specific lncRNA signatures and clarify their association with pathological stage, extranodal extension, and survival outcomes. Such studies would provide a molecular framework for understanding metastatic progression in HNSCC. USgFNAB is a well-established method for cytopathological assessment, yet its molecular diagnostic potential in HNSCC remains untapped. Future research should focus on developing and validating quantitative reverse-transcription PCR (qRT-PCR) or next-generation sequencing (NGS) assays for detecting lncRNAs in FNAB-derived RNA. Comparative analyses between FNAB samples and surgically excised lymph nodes could determine diagnostic concordance, while the identification of minimal, clinically relevant gene panels may facilitate rapid, point-of-care testing. Multiple lncRNAs have been implicated in lymph node metastasis to varying degrees, but their mechanism of action in HNSCC remains incompletely understood. Targeted in vitro and in vivo studies are warranted to identify the molecular pathways through which these lncRNAs modulate invasion, epithelial-to-mesenchymal transition, angiogenesis, and pre-metastatic niche formation. Emerging evidence suggests that primary tumors can prime sentinel lymph nodes for metastatic colonization via the release of soluble factors and extracellular vesicles. This phenomenon has not yet been confirmed in HNSCC. Future studies should evaluate the lncRNA content in extracellular vesicles derived from both primary tumors and draining lymph nodes, and assess their utility as early biomarkers of lymphatic spread. Conventional nodal staging relies on imaging and histopathology, both of which have limited sensitivity for occult metastases. Integrative approaches combining lncRNA expression profiles, mRNA transcriptomics, proteomics, and clinical parameters, analyzed through machine learning algorithms, could significantly improve predictive accuracy. Prospective, multi-center validation of such predictive models will be essential to adopting them in clinical practice. For lncRNA-based biomarkers to impact clinical decision-making, their detection must be feasible in a routine diagnostic setting. The development of rapid, standardized, and cost-effective assays for use on FNAB samples should be prioritized. Prospective clinical trials comparing these molecular diagnostic methods with conventional imaging and cytopathology will be necessary to establish their clinical utility and cost-effectiveness. In summary, the integration of lncRNA-based molecular diagnostics into the current staging paradigm for HNSCC has the potential to improve the preoperative detection of occult nodal metastases, optimize treatment planning, and reduce unnecessary surgical morbidity.

## 9. Conclusions

Accurate preoperative detection of occult cervical lymph node metastases remains a challenge in HNSCC. While USgFNAB provides high specificity, its limited sensitivity restricts reliable identification of micrometastatic disease. Accumulating evidence indicates that dysregulated lncRNAs such as HOTAIR, MALAT1, UCA1, TUG1, AFAP1-AS1, H19, MEG3, and ADAMTS9-AS2 could serve as sensitive and specific biomarkers for metastatic disease. Future research should focus on validating lncRNA panels using FNAB-derived material, establishing standardized assays, and integrating molecular data with clinical and imaging parameters. Successful translation of these approaches could enable more precise risk stratification, reduce unnecessary surgical intervention, and improve patient outcomes.

## Figures and Tables

**Table 1:** Summary of transcriptome studies in head and neck squamous cell carcinoma.

Study Type/Sample Source	Methodology	Key Findings Relevant to Metastasis	Clinical Relevance	Key Reference(s)
Primary tumor vs. normal tissue biopsies	cDNA microarrays; bulk RNA sequencing; TCGA-based transcriptomic analyses	Changes in expression of genes involved in ECM remodeling, angiogenesis, immune responses, and cytoskeletal regulation; prognostic mRNA and lncRNA signatures; immune-related transcripts associated with overall survival	Identification of diagnostic and prognostic biomarkers; molecular stratification of HNSCC	[28,29,30,31,32,33,34,35,36,37]
Primary tumors vs. N0	Bulk transcriptome analysis (RNA-seq, microarrays); integrative bioinformatics	Distinct transcriptional profiles associated with lymphatic spread; overexpression of actin-related genes (e.g., ACTA1); Raf-1 identified as a molecular risk factor for lymphatic metastasis	Prediction of metastatic risk; identification of early markers of nodal involvement	[39,40,41,42]
Primary tumor biopsies vs. matched lymph node metastases	Microarray analysis; next-generation RNA sequencing	Subtype-specific differences between primary tumors and LNMs; immune, invasive, and metabolic/proliferative LNM subtypes; invasive subtype associated with poor prognosis	LNMs as a source of prognostic molecular information	[41]
Single-cell transcriptomics of primary tumors and LNMs	Single-cell RNA sequencing (scRNA-seq)	Partial epithelial-to-mesenchymal transition (p-EMT) programs; localization of p-EMT cells at tumor margins associated with nodal metastasis and adverse pathological features	High-resolution identification of metastatic cell populations and predictive cellular signatures	[42]
FNAB lymph node aspirates	Targeted gene expression analysis (qRT-PCR); no RNA-seq studies to date	Limited evidence; feasibility of targeted gene and miRNA detection demonstrated in other malignancies	Underexplored diagnostic material with potential for transcriptomic biomarker development	[44]
Brush biopsy samples	Targeted RNA assays; cytology; epigenomic and proteomic profiling	High diagnostic accuracy for SCC and HPV detection; feasibility of methylomic and proteomic profiling; dysregulated pathways related to cell adhesion, cytoskeletal organization, and immune responses	Minimally invasive sampling for multi-omics biomarker discovery and disease surveillance	[46,47,48,49,50,51]

**Table 2:** lncRNAs associated with head and neck squamous cell carcinoma.

lncRNA	Tumor Subtypes	Sample Type	Study Size (n)	Mechanism of Action	Association with LN Status	Diagnostic/Prognostic Metrics	Independent Predictor	Key Reference(s)
HOTAIR	OSCC, LSCC, NPC	Tumor tissue	6–900	Epigenetic silencing via PCR2 (EZH2)- and LSD1-mediated chromatin remodeling; induction of epithelial–mesenchymal transition through repression of E-cadherin and upregulation of mesenchymal markers; competing endogenous RNA activity via miRNA sponging	High expression correlated with LN metastasis and advanced stage	AUC: 0.81–0.88; sensitivity: up to 82%	Yes	[60,61,62,63,64,65]
MALAT1	OSCC, LSCC, NPC	Tissue, serum	~40	Regulation of chromatin modification and transcription; ceRNA promotes proliferation and metastasis	Overexpression associated with LN+ disease	Association with DFS and OS reported	Partially	[66,67,68]
UCA1	Tongue SCC	Tumor tissue	94	Promotion of tumor cell migration and invasiveness via ceRNA-related pathways	Higher levels in LN+ tumors (*p* = 0.371)	No formal metrics reported	No	[69]
TUG1	OSCC, LSCC	Tumor tissue	64	Enhancement of metastatic potential via miRNA sponging and downstream signaling	Upregulated in LN+ disease	Limited metrics	No	[70,71]
AFAP1-AS1	OSCC, LSCC, NPC	Tissue, serum	60–110	Regulation of cytoskeletal dynamics; promotion of proliferation and lymphatic metastasis	Elevated in tumors with lymphatic spread	Serum biomarker AUC: ~0.79	No	[67,72,73]
H19	OSCC, NPC	Tumor tissue	6–16	Oncogenic ceRNA; promotion of migration and invasion via miR-138/let-7 sponging	Higher expression in LN+ patients	AUC 0.75–0.83	Yes	[74,75,76]
MEG3	OSCC	Tumor tissue	45–83	Tumor-suppressive lncRNA inhibiting migration and metastasis via miRNA and WNT/β-catenin pathways	Low expression associated with metastasis	Linked to worse OS and DFS	Yes	[77,78]
ADAMTS9-AS2	Tongue SCC	Tumor tissue	76	Promotion of tumor growth, migration, invasion, and EMT	Overexpression correlated with nodal disease	Limited metrics	Partially	[79]

## Data Availability

No new data were created or analyzed in this study.

## Abbreviations

The following abbreviations are used in this manuscript:

HNSCC	head and neck squamous cell carcinoma
FNAB	fine-needle aspiration biopsy
USgFNAB	ultrasound-guided fine-needle aspiration biopsy
lncRNAs	long non-coding RNAs
AJCC	American Joint Committee on Cancer
SLNB	sentinel lymph node biopsy
HPV	human papilloma virus
ECM	extracellular matrix
TCGA	The Cancer Genome Atlas
LN	lymph node
PNI	perineural invasion
LNM	lymph node metastasis
p-EMT	partial epithelial-to-mesenchymal transition
ncRNA	non-coding RNA
lncRNA	long non-coding RNA
tRNA	transfer RNA
rRNA	ribosomal RNA
snRNA	small nuclear RNA
snoRNA	small nucleolar RNA
siRNA	small interfering RNA
aslncRNA	antisense lncRNA
eRNA	enhancer RNA
HOTAIR	HOX antisense intergenic RNA
EZH2	enhancer of zeste homolog 2
PRC2	polycomb repressive complex 2
LSD1	lysine-specific demethylase 1
ceRNA	competing endogenous RNA
MTA2	metastasis-associated gene 2
SNPs	single-nucleotide polymorphisms
OSCC	oral squamous cell carcinoma
MALAT1	Metastasis-Associated Lung Adenocarcinoma Transcript 1
UCA1	urothelial cancer-associated 1
TUG1	taurine upregulated gene 1
AFAP1-AS1	actin filament-associated protein 1 antisense RNA1
MEG3	maternally expressed 3
ADAMTS9-AS2	ADAM metallopeptidase with thrombospondin type 1 motif, 9 antisense RNA 2
qRT-PCR	quantitative reverse-transcription PCR
NGS	next-generation sequencing
